# Supplementary material for: Economic impact of a machine learning-based strategy for preparation of blood products in brain tumor surgery
Source: PLoS One. 2022 Jul 1;17(7):e0270916. doi: 10.1371/journal.pone.0270916 (PMC9249218; doi:10.1371/journal.pone.0270916)
Supplement: S1 File — (DOCX) [file pone.0270916.s001.docx]

**Supplement**

**Economic Impact of Machine Learning-Based Strategy for Blood Preparation in Brain Tumor Surgery**

**Methods**

The processes of the present study comprised two major sections as follows: 11) Development of the predictive models and deployment of the machine learning (ML)-based web application, and 2) validation of the web application.

**1. Development of the predictive models and deployment of the machine learning (ML)-based web application**

Total data in the present study were divided into two cohorts that were collected from patients with a brain tumor who had undergone cranial operation between January 2014 and December 10, 2021 at this single-center hospital were included. In detail, the first cohort dataset was collected from patients during January 2014 and December 2019 that was divided into a training dataset and testing dataset by 70:30 randomly splitting methods, as shown in Figure S1.


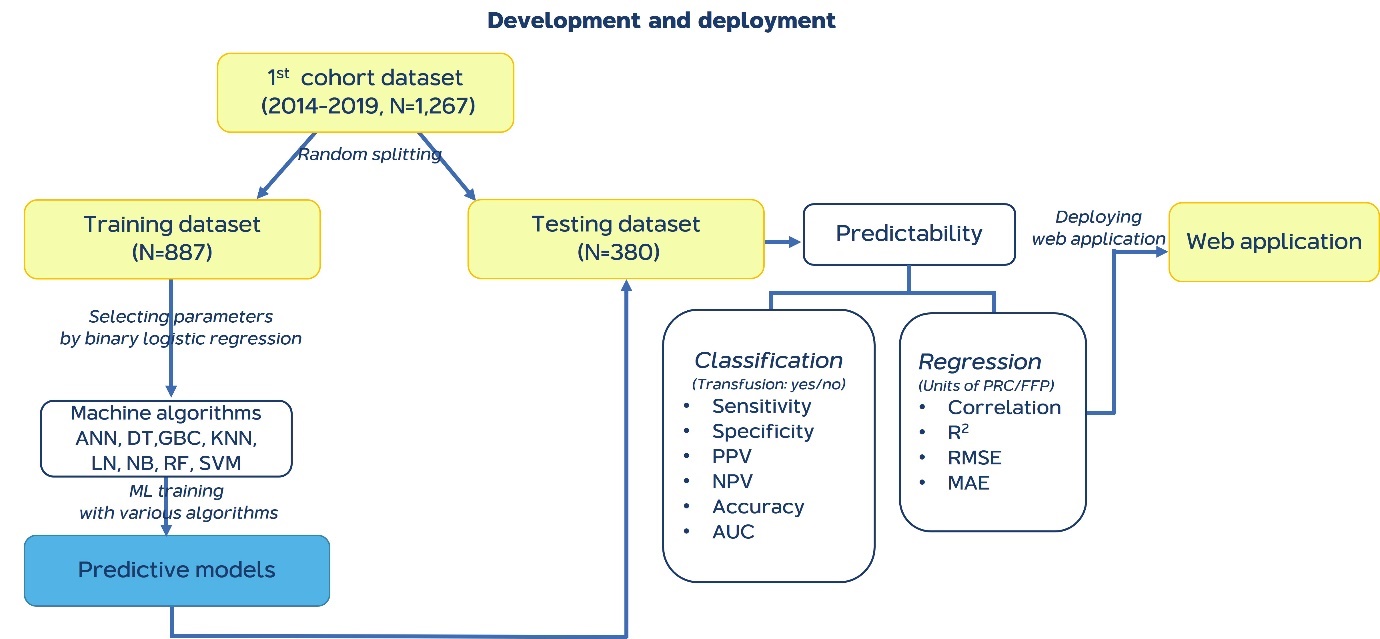


**Figure S1. Workflow of development and deployment of the ML-based web application.**

Baseline characteristics and preoperative hematologic laboratories of the 1^st^ cohort with 1,267 patients are presented in Table S1.

**Table S1. Baseline characteristics of 1^st^ cohort (2014-2019, N=1,267)**

| **Characteristics** | **Total (%)** |
| --- | --- |
| **Sex** |  |
| Male | 540 (42.6) |
| Female | 727 (57.4) |
| **Mean age-year (SD)** | 47.76 (17.49) |
| **Age-year** |  |
| 0-15 | 103 (8.1) |
| >15-60 | 883 (69.7) |
| >60 | 281 (22.2) |
| **Underlying disease** |  |
| Hypertension | 196 (15.5) |
| Diabetes mellitus | 134 (12.6) |
| Dyslipidemia | 133 (10.5) |
| Liver disease | 22 (1.7) |
| Renal failure | 30 (2.4) |
| Preoperative seizure | 116 (9.2) |
| **Preoperative current medication** |  |
| Antiplatelet | 18 (1.4) |
| Clexane | 3 (0.2) |
| Warfarin | 2 (0.2) |
| **American Society of Anesthesiologists classification** |  |
| 1 | 2 (0.2) |
| 2 | 171 (13.5) |
| 3 | 1089 (86.0) |
| 4 | 5 (0.4) |
| **Tumor classification** |  |
| Meningioma | 474 (37.4) |
| Glioma | 371 (29.3) |
| Pituitary adenoma | 168 (13.3) |
| Schwannoma | 81 (6.4) |
| Metastasis | 64 (5.1) |
| Lymphoma | 13 (1.0) |
| Other | 96 (7.6) |
| **Mean diameter of tumor -cm (SD)** | 3.12 (0.84) |
| **Mean preoperative midline shift -cm (SD)** | 0.42 (0.84) |
| **Neurosurgical operation** |  |
| Craniotomy | 712 (56.2) |
| Craniectomy | 79 (6.2) |
| Suboccipital or, rectosigmoid approach | 169 (13.3) |
| Endoscopic transsphenoidal approach | 173 (13.7) |
| Burr hole with biopsy | 125 (9.9) |
| Endoscopic third ventriculostomy with biopsy | 9 (0.7) |
| **Emergency operation** | 87 (6.9) |
| **Mean body mass index- kg/m^2^** | 23.58 (4.55) |
| **Mean preoperative hematocrit-%** | 39.27 (14.52) |
| **Mean preoperative hemoglobin-** **g/dL** | 12.87 (1.72) |
| **Mean platelet count- x10^3^/µL** | 285.11 (91.24) |
| **Mean white blood cell count- x10^3^/µL** | 9.84 (4.49) |
| **Mean neutrophil /lymphocyte ratio** | 5.02 (7.09) |
| **Mean partial thromboplastin time ratio** | 0.93 (0.13) |
| **Mean international normalized ratio** | 1.14 (3.40) |

Using the training dataset, binary logistic regression was analyzed for estimating factors associated with intraoperative transfusion in both univariate and multivariable analyses, as shown in Table S2. From multivariable analysis with the backward stepwise procedure, the factors were significantly associated with intraoperative transfusion as follows: gender, hypertension, preoperative seizure, American Society of Anesthesiologists classification, tumor classification, a diameter of tumor, body mass index, hemoglobin, and operation.

**Table S2. Binary logistic regression analysis for intracranial injury by the training dataset (N=887)**

|  | **Univariate analysis** |  | **Multivariable analysis** |  |
| --- | --- | --- | --- | --- |
| **Factor** | **Odds ratio (95%CI)** | **p-value** | **Odds ratio (95%CI)** | **p-value** |
| **Gender** |  |  |  |  |
| Male | Ref |  | Ref |  |
| Female | 1.92 (1.45-2.48) | <0.001 | 1.48 (1.05-2.10) | 0.02 |
| **Age group** |  |  |  |  |
| 0-15 | Ref |  |  |  |
| >15-60 | 0.68 (0.44-1.05) | 0.08 |  |  |
| >60 | 0.69 (0.42-1.14) | 0.15 |  |  |
| **Underlying disease** |  |  |  |  |
| Hypertension* | 2.79 (2.00-3.77) | <0.001 | 3.62 (2.41-5.43) | <0.001 |
| Diabetes mellitus* | 0.76 (0.50-1.15) | 0.20 |  |  |
| Dyslipidemia* | 2.22 (1.53-3.22) | <0.001 |  |  |
| Liver disease* | 1.65 (0.68-3.97) | 0.26 |  |  |
| Renal failure* | 2.57 (1.24-5.33) | 0.01 |  |  |
| Preoperative seizure * | 2.37 (1.60-3.51) | <0.001 | 1.82 (1.06-3.11) | 0.02 |
| **Preoperative current medication** |  |  |  |  |
| Antiplatelet * | 1.43 (0.53-3.86) | 0.47 |  |  |
| Warfarin * | 2.86 (0.17-45.99) | 0.45 |  |  |
| **American Society of Anesthesiologists classification** |  |  |  |  |
| 1-2 | Ref |  | Ref |  |
| 3-4 | 1.95 (1.27-2.99) | 0.002 | 3.62 (2.41-5.43) | <0.001 |
| **Tumor classification** |  |  |  |  |
| Meningioma | Ref |  | Ref |  |
| Glioma | 0.29 (0.21-0.40) | <0.001 | 0.36 (0.23-0.56) | <0.001 |
| Pituitary adenoma | 0.22 (0.13-0.36) | <0.001 | 0.84 (0.22-3.15) | 0.80 |
| Schwannoma | 0.41 (0.23-0.72) | 0.02 | 0.39 (0.18-0.85) | 0.01 |
| Metastasis | 0.35 (0.18-0.68) | 0.02 | 0.24 (0.10-0.57) | 0.001 |
| Lymphoma | 0.12 (0.01-1.00) | 0.05 | 0.23 (0.02-2.30) | 0.21 |
| Other | 0.77 (0.48-1.23) | 0.27 | 0.64 (0.34-1.21) | 0.17 |
| **Diameter of tumor-cm** | 2.14 (1.81-2.54) | <0.001 | 2.16 (1.77-2.64) | <0.001 |
| **Preoperative midline shift-cm** | 2.16 (1.08-4.30) | 0.02 |  |  |
| **Emergency operation*** | 0.67 (0.39-1.17) | 0.16 |  |  |
| **Body mass index- kg/m^2^** | 0.94 (0.92-0.97) | 0.001 | 0.88 (0.85-0.92) | <0.001 |
| **Preoperative hematologic laboratory** |  |  |  |  |
| Hematocrit-% | 0.68 (0.62-0.75) | <0.001 |  |  |
| Hemoglobin- g/dL | 0.91 (0.88-0.94) | <0.001 | 0.54 (0.48-0.62) | <0.001 |
| Platelet count- x10^3^/µL | 1.00 (0.99-1.002) | 0.33 |  |  |
| White blood cell count- x10^3^/µL | 1.01 (0.98-1.05) | 2.92 |  |  |
| Neutrophil /lymphocyte ratio | 1.01(0.99-1.03) | 0.18 |  |  |
| Partial thromboplastin time ratio | 0.89 (0.30-2.60) | 0.83 |  |  |
| International normalized ratio | 0.96 (0.85-1.09) | 0.57 |  |  |
| **Neurosurgical operation** |  |  |  |  |
| Craniotomy | Ref |  | Ref |  |
| Craniectomy | 0.76 (0.45-1.28) | 0.76 | 0.69 (0.36-1.32) | 0.27 |
| Suboccipital/ rectosigmoid approach | 0.68 (0.46-0.99) | 0.04 | 1.27 (0.72-2.25) | 0.40 |
| Endoscopic transsphenoidal approach | 0.26 (0.15-0.42) | <0.001 | 0.20 (0.05-0.74) | 0.01 |
| Burr hole with biopsy | 0.06 (0.02-0.18) | <0.001 | 0.79 (0.02-0.25) | <0.001 |
| Endoscopic third ventriculostomy with biopsy | 0.24 (0.03-2.00) | 0.19 | 0.70 (0.005-0.93) | 0.04 |
| * Data show only “yes group” while reference groups (no group) are hidden. | | | | |

Therefore, various ML algorithms were trained with the selected factors associated with transfusion as a prior process both classification and regression. The ML classification was performed for predicting binary classifiers (transfusion or no transfusion), while the ML regression was done for forecasting the number of packed red cell (PRC) and fresh frozen plasma (FFP) units which should be intraoperatively used.

The supervised ML algorithms were used for classification with a 5-fold cross-validation process as follows: naïve Bayes (NB), artificial neural network (ANN), support vector machine (SVM), k-nearest neighbors (KNN), decision tree (DT), random forest (RF), and gradient boosting classifier (GBC). While algorithms of the ML were linear regression, ANN, DT, RF, and GBC.

Then, sensitivity, specificity, positive predictive value, negative predictive value, accuracy, and area under the receiver operating characteristic curve (AUC) were validated for the evaluation of the predictability using the testing dataset. The performance of the predictability of ML classification was revealed for intraoperative PRC transfusion. For regression, correlation with scatter plot, R2, root mean squared error (RMSE) and mean absolute error (MAE) were calculated for estimating the performance of ML regression. Table S3 shows the predictability of classification and regression using the testing dataset and the receiver operating characteristic (ROC) curves with AUCs of classification are shown in Figure S2.

**Table S3. Predictability of ML for intraoperative PRC transfusion by testing dataset (N=380)**

| **Algorithm** | **Sensitivity** | **Specificity** | **PPV** | **NPV** | **Accuracy** | **AUC** |
| --- | --- | --- | --- | --- | --- | --- |
| **Classification** | | | | | | |
| Random  forest | 0.70  (0.61-0.70) | 0.92  (0.89-0.95) | 0.75  (0.66-0.84) | 0.90  (0.86-0.93) | 0.86  (0.83-0.90) | 0.82  (0.75-0.87) |
| Gradient boosting classifier | 0.68  (0.59-0.78) | 0.93  (0.90-0.96) | 0.78  (0.69-0.87) | 0.89  (0.86-0.93) | 0.87  (0.84-0.90) | 0.81  (0.75-0.87) |
| Decision  tree | 0.67  (0.58-0.77) | 0.91  (0.88-0.94) | 0.73  (0.63-0.82) | 0.89  (0.85-0.92) | 0.85  (0.76-0.82) | 0.80  (0.73-0.85) |
| naïve  Bayes | 0.55  (0.45-0.65) | 0.94  (0.91-0.97) | 0.76  (0.68-0.86) | 0.86  (0.82-0.90) | 0.84  (0.80-0.88) | 0.75  (0.68-0.81) |
| Artificial neural network | 0.47  (0.37-0.57) | 0.91  (0.87-0.94) | 0.64  (0.53-0.75) | 0.83  (0.79-0.87) | 0.80  (0.69-0.75) | 0.70  (0.62-0.76) |
| Support vector machine | 0.43  (0.33-0.53) | 0.94  (0.91-0.97) | 0.72  (0.60-0.83) | 0.83  (0.79-0.  87) | 0.81  (0.77-0.85) | 0.69  (0.62-0.75) |
| k-nearest  neighbors | 0.33  (0.23-0.42) | 0.94  (0.91-0.96) | 0.65  (0.51-0.78) | 0.80  (0.76-0.84) | 0.78  (0.74-0.82) | 0.64  (0.56-0.70) |
| **Regression** | | | | | | |
|  | **Pearson’s correlation** | **p-value of Pearson’s correlation** | **Spearman’s rank correlation** | **R^2^** | **RMSE** | **MAE** |
| Random forest | 0.80 | <0.001 | 0.61 | 0.63 | 1.06 | 0.54 |
| Decision tree | 0.80 | <0.001 | 0.62 | 0.64 | 1.05 | 0.55 |
| Gradient boosting classifier | 0.76 | <0.001 | 0.56 | 0.57 | 1.14 | 0.59 |
| k-nearest neighbors | 0.62 | <0.001 | 0.48 | 0.80 | 1.38 | 0.64 |
| Linear regression | 0.60 | <0.001 | 0.38 | 0.35 | 1.41 | 0.91 |
| Artificial neural network | 0.41 | <0.001 | 0.41 | 0.51 | 1.22 | 0.71 |
| Abbreviations: AUC= Area under the ROC curve, NPV=negative predictive value, PPV=positive predictive value, PRC=packed red cell, R^2^= R-squared, RF= random forest classifier, RMSE= Root Mean Squared Error, MAE= Mean Absolute Error, ML=machine learning | | | | | | |


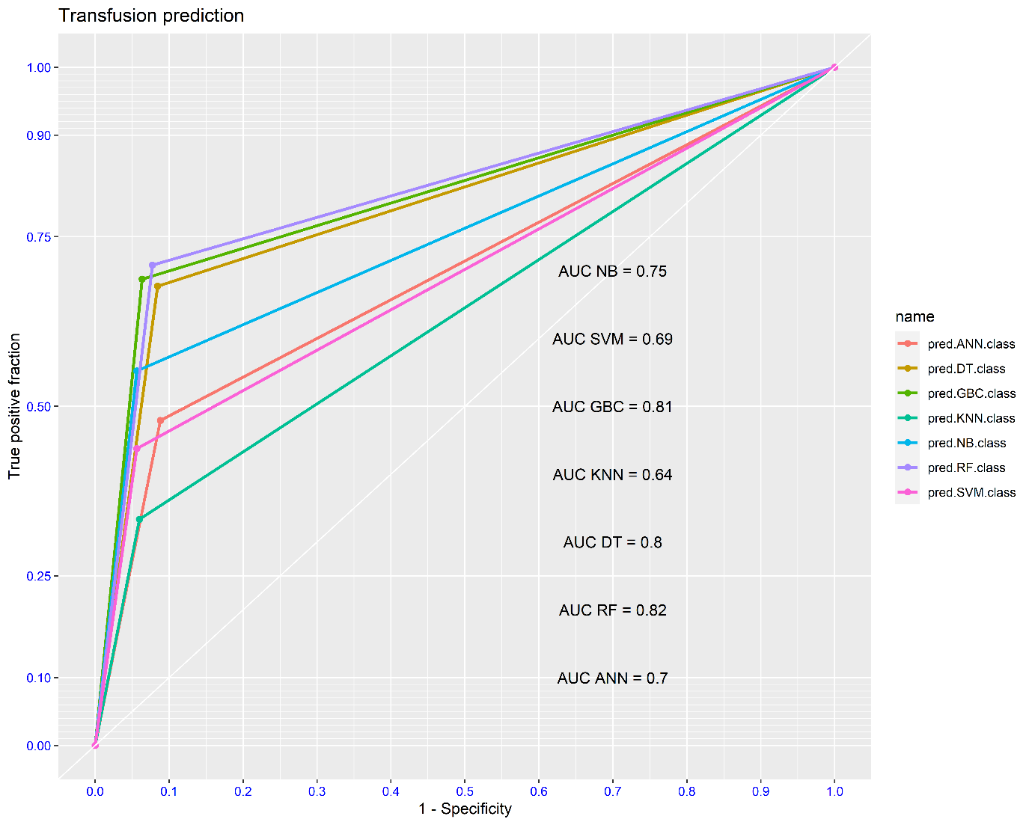


**Figure S2. The receiver operating characteristic curves with area under the curve of classification using testing dataset. Abbreviations: ANN= artificial neural network, DT= decision tree, GBC= Gradient boosting classifier, KNN= k-nearest neighbors, NB= naïve Bayes, SVM= Support vector machine**

For intraoperative PRC prediction, scatter plots of various regression algorithms are presented as shown in Figure S3. The RF algorithm was the best performance of prediction both classification and regression.


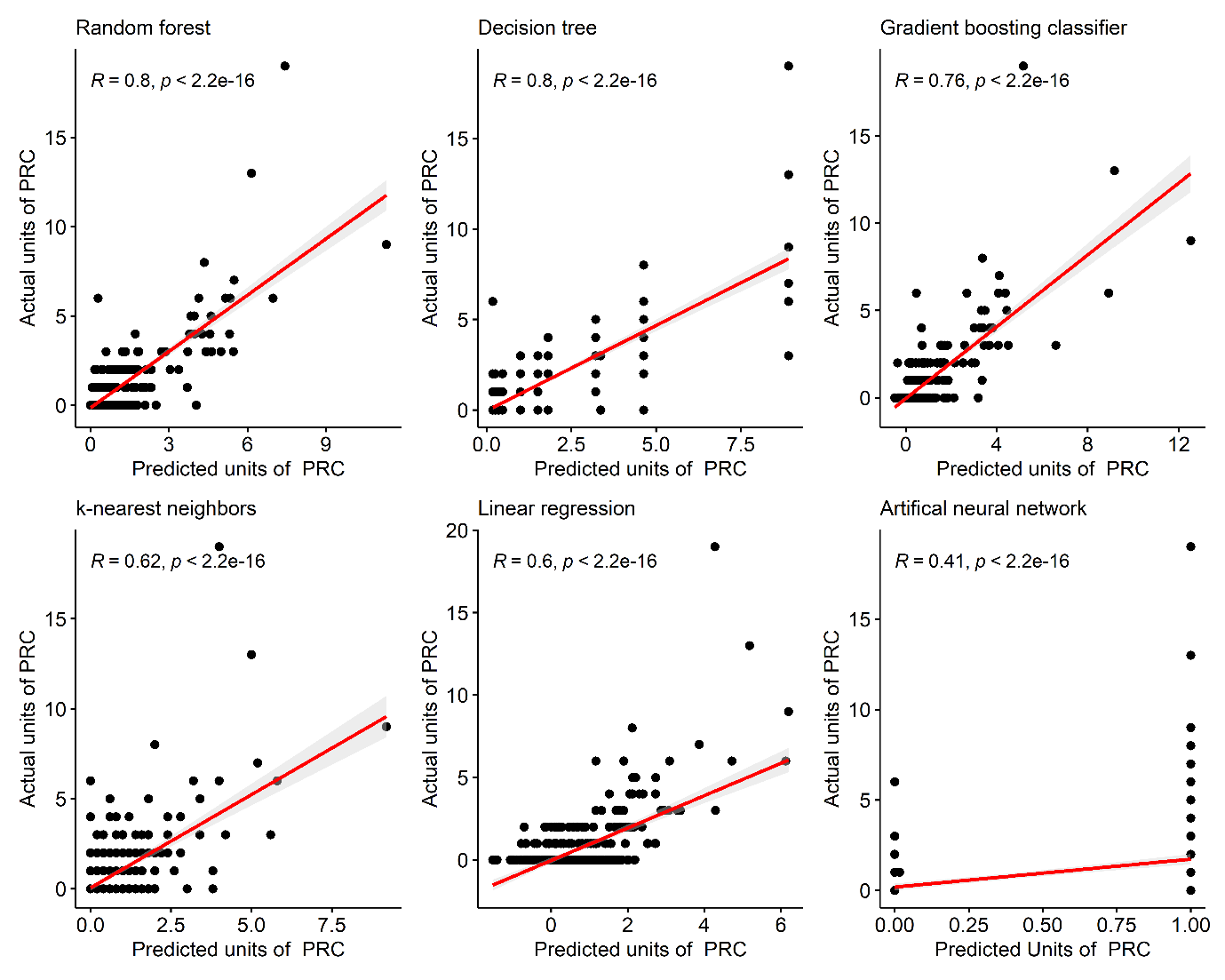


**Figure S3. Scatter plots of various regression algorithms for prediction of units of PRC using testing dataset. Abbreviation: PRC= packed red cell, R= Pearson correlation.**

Next, simple linear regressions were performed for evaluating factors associated with units of FFP transfusion. Results of simple linear regression analysis are shown in Table S4.

**Table S4. Simple linear regression analysis for the number of units of FFP transfusion by training dataset (N=887)**

| **Factor** | **Constant** | **Beta-coefficient**  **(95%CI)** | **Odds ratio (95%CI)** |
| --- | --- | --- | --- |
| **Gender (female)** | 0.20 | 0.29 (0.08,0.49) | 0.006 |
| **Age** | 0.50 | 0.003 (-0.003,0.009) | 0.28 |
| **Underlying disease** |  |  |  |
| Hypertension* | 0.58 | 0.52 (0.24-0.80) | <0.001 |
| Diabetes mellitus* | 0.81 | -0.17 (-0.53,0.19) | 0.36 |
| Dyslipidemia* | 0.63 | 0.25(-0.08,0.58) | 0.14 |
| Liver disease* | 0.65 | 0.75 (-0.28,1.54) | 0.059 |
| Renal failure* | 0.63 | 1.19(0.52,1.87) | 0.001 |
| Preoperative seizure * | 0.60 | 0.65 (0.30-1.01) | <0.001 |
| **Preoperative current medication** |  |  |  |
| Antiplatelet * | 0.66 | 0.22(-0.64,1.10) | 0.60 |
| Warfarin * | 0.66 | -0.66(-3.25,1.93) | 0.61 |
| **American Society of Anesthesiologists classification (Class3-4)** | 0.45 | 0.24 (-0.05,0.54) | 0.10 |
| **Tumor classification** |  |  |  |
| Meningioma | 0.36 | 0.80 (0.59,1.01) | <0.001 |
| Glioma | 0.76 | -0.35 (-0.57, -0.12) | 0.002 |
| Pituitary adenoma | 0.74 | -0.63 (-0.93, -0.33) | <0.001 |
| Schwannoma | 0.69 | -0.43 (-0.85, -0.01) | 0.04 |
| Metastasis | 0.68 | -0.45 (-0.92, 0.01) | 0.06 |
| Lymphoma | 0.67 | -0.67 (-1.69, 0.35) | 0.19 |
| **Diameter of tumor-cm** | -2.68 | 1.07 (0.96,1.17) | <0.001 |
| **Preoperative midline shift-cm** | 0.07 | 1.31 (0.73,1.90) | <0.001 |
| **Emergency operation*** | 0.64 | 0.23 (-0.16,0.64) | 0.25 |
| **Body mass index- kg/m^2^** | 1.07 | -0.01 (-0.04,0.007) | 0.16 |
| **Preoperative hematologic laboratory** |  |  |  |
| Hematocrit-% | 0.87 | -0.005 (-0.01,0.004) | 0.24 |
| Hemoglobin- g/dL | 1.70 | -0.08 (-0.15,-0.006) | 0.03 |
| Platelet count- x10^3^/µL | 1.58 | -0.002 (-0.003,0.00) | 0.11 |
| White blood cell count- x10^3^/µL | 0.76 | 0.04 (0.002,0.07) | 0.03 |
| Neutrophil /lymphocyte ratio | 1.04 | 0.02 (-0.003, 0.04) | 0.07 |
| Partial thromboplastin time ratio | 2.23 | -1.30 (-2.55,-0.05) | 0.04 |
| International normalized ratio | 1.16 | -0.01 (-0.06,0.38) | 0.62 |
| **Neurosurgical operation** |  |  |  |
| Craniotomy | 0.35 | 0.55 (0.35,0.76) | <0.001 |
| Craniectomy | 0.63 | 0.41 (-0.01,0.83) | 0.057 |
| Suboccipital/ rectosigmoid approach | 0.69 | -0.26 (-0.56,0.04) | 0.09 |
| Endoscopic transsphenoidal approach | 0.73 | -0.56 (-0.85,-0.26) | <0.001 |
| Burr hole with biopsy | 0.73 | -0.69 (-1.03,-0.34) | <0.001 |
| Endoscopic third ventriculostomy with biopsy | 0.66 | -0.55 (-1.78,0.67) | 0.37 |
| **Number of intraoperative PRC transfusion-unit** | 0.59 | 1.09 (1.03,1.15) | <0.001 |
| Abbreviations: FFP= Fresh frozen plasma, PRC=packed red cell | | | |

Hence, multiple linear regression was analyzed with a stepwise backward elimination procedure and results are shown in Table S5.

**Table S5. Multiple linear regression analysis for the number of units of FFP transfusion by training dataset (N=887)**

| **Factor** | **Beta-coefficient (95%CI)** | **p-value** |
| --- | --- | --- |
| **Constant** | **-2.54** |  |
| **Preoperative hemoglobin**- g/dL | 0.25 (0.14,0.26) | <0.001 |
| **Number of intraoperative PRC transfusion-unit** | 0.91 (0.87,0.96) | <0.001 |
| Abbreviations: FFP= Fresh frozen plasma, PRC=packed red cell | | |

Using ML regression for intraoperative FFP transfusion, results are shown in Table S6.

**Table S6. Predictability of ML regression for intraoperative FFP transfusion by testing dataset (N=380)**

| **Algorithm** | **Pearson’s correlation** | **p-value of Pearson’s correlation** | **Spearman’s rank correlation** | **R^2^** | **RMSE** | **MAE** |
| --- | --- | --- | --- | --- | --- | --- |
| Linear regression | 0.80 | <0.001 | 0.49 | 0.64 | 1.20 | 0.56 |
| Gradient boosting classifier | 0.80 | <0.001 | 0.46 | 0.63 | 1.21 | 0.58 |
| Artificial neural network | 0.79 | <0.001 | 0.46 | 0.62 | 1.23 | 0.54 |
| Decision tree | 0.79 | <0.001 | 0.57 | 0.62 | 1.24 | 0.56 |
| Random forest | 0.77 | <0.001 | 0.48 | 0.59 | 1.28 | 0.55 |
| k-nearest neighbors | 0.78 | <0.001 | 0.49 | 0.63 | 1.23 | 0.54 |
| Abbreviations: FFP= fresh frozen plasma, R^2^= R-squared, RF= random forest classifier, RMSE= Root Mean Squared Error, MAE= Mean Absolute Error, ML=machine learning | | | | | | |

Comparison of intraoperative FFP predictability among various regression algorithms was performed using scatter plots, as shown in Figure S4. The linear regression algorithm was the best performance of intraoperative FFP predictability


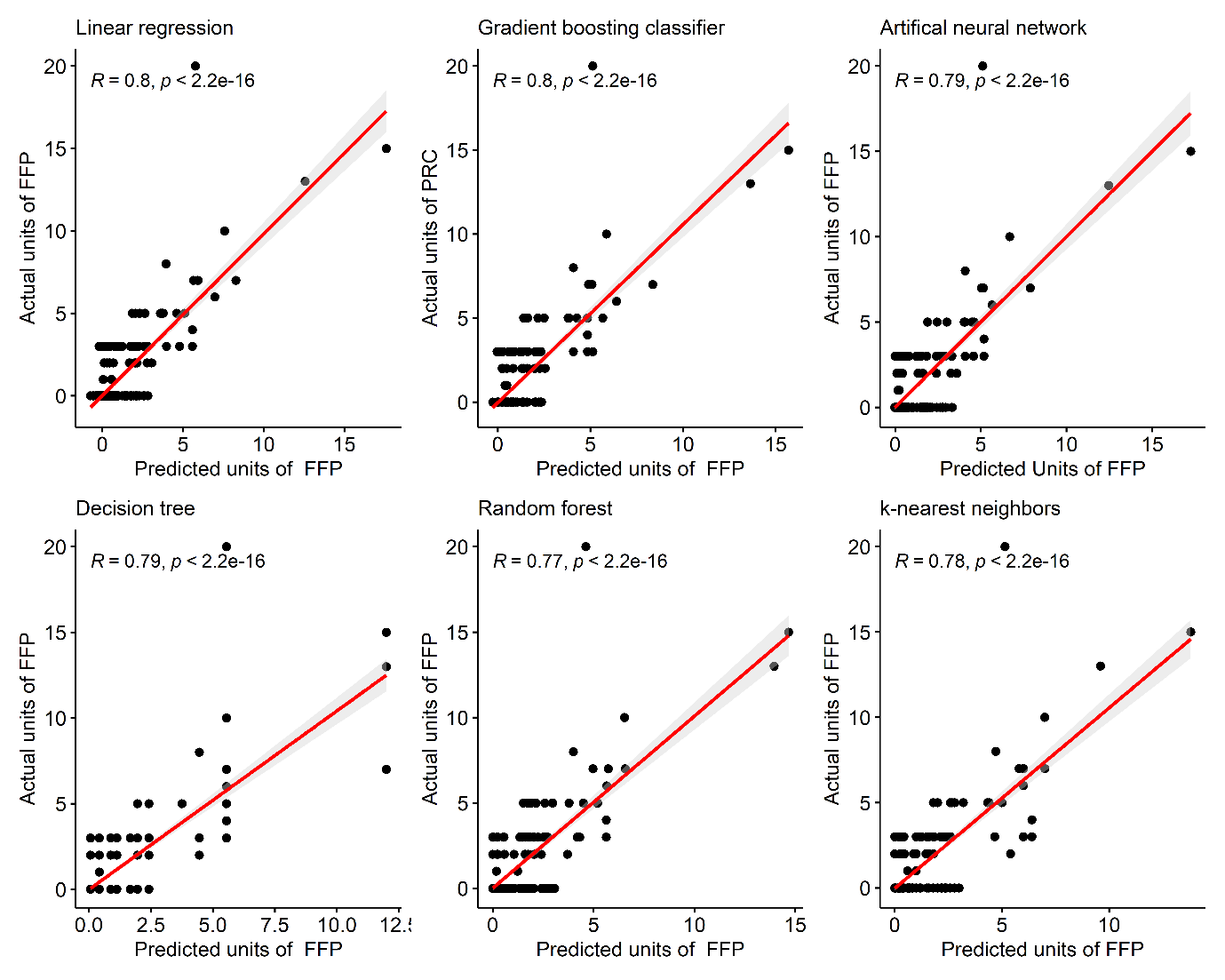


**Figure S4. Scatter plots of various regression algorithms for the prediction of units of FFP. Abbreviation: FFP= Fresh frozen plasma, R= Pearson correlation.**

As the results, RF and linear regression algorithms were used and deployed as the ML-based web application for the prediction of units of PRC and FFP, respectively.

Statistical analysis was performed using R version 4.0.5 (The R Foundation for Statistical Computing; Vienna, Austria) and ML was performed with the “caret” package. Moreover, the ML-based web application was developed and deployed using "shiny" package. Additionally, the code of ML can be found on GitHub (GitHub, Inc.: <https://github.com/Thara-PSU/ML_transfusion_EE> ).

**2. Validation of the ML-based web application**

Because overfitting performance of predictive models was concerned, we validated these models from unseen data as the second cohort dataset. The dataset was collected from patients who had undergone brain tumor surgery between January 2020 and December 10, 2021, as shown in Figure S8.


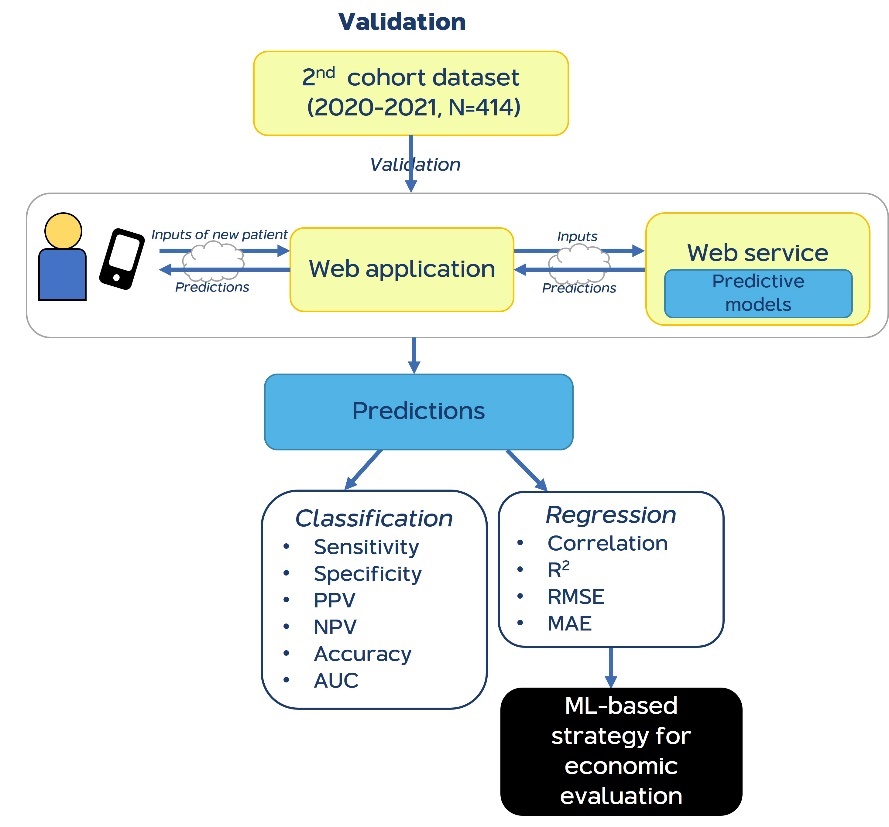


**Figure S8. Workflow validation of the ML-based web application**

Therefore, demographic data of the 2^nd^ cohort are present by intraoperative PRC transfusion, as shown in Table S7.

**Table S7. Baseline characteristics of the 2^nd^ cohort by PRC transfusion (2020-2021, N=414)**

| **Characteristics** | **Transfusion**  **(N=114)** | **No transfusion**  **(N=298)** |
| --- | --- | --- |
| **Sex** |  |  |
| Male | 35 (30.7) | 141 (47.0) |
| Female | 79 (69.3) | 159 (53.0) |
| **Mean age-year (SD)** |  |  |
| **Age-year** |  |  |
| 0-15 | 15 (13.2) | 20 (6.7) |
| >15-60 | 82 (71.2) | 217 (72.3) |
| >60 | 17 (14.9) | 63 (21.0) |
| **Underlying disease** |  |  |
| Hypertension | 29 (25.4) | 49 (16.3) |
| Diabetes mellitus | 14 (12.3) | 25 (8.4) |
| Dyslipidemia | 26 (22.8) | 36 (12.0) |
| Liver disease | 4 (3.5) | 8 (2.7) |
| Renal failure | 1 (0.9) | 4 (1.3) |
| Preoperative seizure | 19 (16.7) | 25 (8.3) |
| **Preoperative current medication** |  |  |
| Antiplatelet | 2 (1.8) | 7 (2.3) |
| Clexane | 1 (0.9) | 3 (1.0) |
| Warfarin | 1 (0.9) | 1 (0.3) |
| **American Society of Anesthesiologists classification** |  |  |
| 1 | 0 | 1 (0.3) |
| 2 | 28 (24.6) | 83 (27.7) |
| 3 | 83 (72.8) | 213 (71.0) |
| 4 | 3 (2.6) | 3 (1.0)_ |
| **Tumor classification** |  |  |
| Meningioma | 69 (60.5) | 89 (29.7) |
| Glioma | 25 (21.9) | 102 (34.0) |
| Pituitary adenoma | 3 (2.6) | 44 (14.7) |
| Schwannoma | 6 (5.3) | 22 (7.3) |
| Metastasis | 4 (3.5) | 21 (7.0) |
| Lymphoma | 0 | 4 (1.3) |
| Other | 7 (6.1) | 18 (6.0) |
| **Mean diameter of tumor -cm (SD)** | 3.55 (1.43) | 2.98 (0.56) |
| **Mean preoperative midline shift -cm (SD)** | 0.59 (0.53) | 0.39 (0.12) |
| **Neurosurgical operation** |  |  |
| Craniotomy | 75 (65.8) | 153 (51.0) |
| Craniectomy | 15 (13.2) | 24 (8.0) |
| Suboccipital or rectosigmoid approach | 18 (15.8) | 35 (11.7) |
| Endoscopic transsphenoidal approach | 4 (3.5) | 47 (15.7) |
| Burr hole with biopsy | 1 (0.9) | 36 (12.0) |
| Endoscopic third ventriculostomy with biopsy | 1 (0.9) | 5 (1.7) |
| **Emergency operation** | 18 (15.8) | 47 (15.7) |
| **Mean body mass index- kg/m^2^** | 23.43 (4.57) | 24.21 (4.67) |
| **Mean preoperative hematocrit-%** | 37.10 (5.19) | 39.59 (4.22) |
| **Mean preoperative hemoglobin-** **g/dL** | 12.22 (1.79) | 13.08 (1.49) |
| **Mean platelet count- x10^3^/µL** | 295.04 (95.59) | 287.18 (87.44) |
| **Mean white blood cell count- x10^3^/µL** | 9.94 (4.55) | 9.85 (4.72) |
| **Mean neutrophil /lymphocyte ratio** | 24.79 (12.27) | 24.83 (13.18) |
| **Mean partial thromboplastin time ratio** | 0.92 (0.12) | 0.93 (0.14) |
| **Mean international normalized ratio** | 1.03 (0.87) | 1.01 (0.87) |

Several ML algorithms were validated by the 2^nd^ cohort for intraoperative PRC transfusion both classification and regression, as shown in Table S8.

**Table S8. Predictability of ML for intraoperative PRC transfusion using the 2^nd^ cohort (N=414)**

| **Algorithm** | **Sensitivity** | **Specificity** | **PPV** | **NPV** | **Accuracy** | **AUC** |
| --- | --- | --- | --- | --- | --- | --- |
| **Classification** | | | | | | |
| Random  forest | 0.70  (0.61-0.79) | 0.92  (0.89-0.95) | 0.75  (0.66-0.84) | 0.90  (0.86-0.93) | 0.86  (0.83-0.90) | 0.73  (0.71-0.75) |
| Gradient boosting classifier | 0.68  (0.59-0.78) | 0.58  (0.54-0.63) | 0.25  (0.20-0.31) | 0.89  (0.86-0.93) | 0.60  (0.56-0.64) | 0.71  (0.68-0.73) |
| Decision  tree | 0.67  (0.58-0.77) | 0.91  (0.88-0.94) | 0.73  (0.63-0.82) | 0.89  (0.85-0.92) | 0.85  (0.81-0.89) | 0.66  (0.64-0.68) |
| naïve  Bayes | 0.55  (0.45-0.65) | 0.94  (0.91-0.97) | 0.76  (0.66-0.86) | 0.86  (0.82-0.90) | 0.84  (0.80-0.88) | 0.73  (0.68-0.78) |
| Artificial neural network | 0.47  (0.37-0.57) | 0.91  (0.87-0.94) | 0.64  (0.53-0.75) | 0.83  (0.79-0.87) | 0.80  (0.76-0.84) | 0.66  (0.62-0.70) |
| Support vector machine | 0.43  (0.33-0.53) | 0.94  (0.91-0.97) | 0.72  (0.60-0.83) | 0.83  (0.79-0.87) | 0.81  (0.77-0.85) | 0.63  (0.60-0.66) |
| k-nearest  neighbors | 0.33  (0.23-0.42) | 0.80  (0.76-0.84) | 0.33  (0.23-0.42) | 0.80  (0.76-0.84) | 0.70  (0.65-0.74) | 0.59  (0.55-0.63) |
| **Regression** | | | | | | |
|  | **Pearson’s correlation** | **p-value of Pearson’s correlation** | **Spearman’s rank correlation** | **R^2^** | **RMSE** | **MAE** |
| Random forest | 0.80 | p<0.001 | 0.40 | 0.54 | 1.35 | 0.79 |
| Decision tree | 0.80 | p<0.001 | 0.36 | 0.12 | 1.99 | 0.99 |
| Gradient boosting classifier | 0.76 | p<0.001 | 0.33 | 0.09 | 2.07 | 1.01 |
| k-nearest neighbors | 0.62 | p<0.001 | 0.27 | 0.07 | 2.04 | 0.97 |
| Linear regression | 0.60 | p<0.001 | 0.37 | 0.47 | 1.49 | 0.89 |
| Artificial neural network | 0.41 | p<0.001 | 0.39 | 0.59 | 1.29 | 0.74 |
| Abbreviations: AUC= Area under the ROC curve, NPV=negative predictive value, PPV=positive predictive value, PRC=packed red cell, R^2^= R-squared, RF= random forest classifier, RMSE= Root Mean Squared Error, MAE= Mean Absolute Error, ML= machine learning | | | | | | |

As the result, the predictability of intraoperative PRC transfusion was slightly dropped in all algorithms, but the random forest still had the lowest RMSE and MSE when the model was validated with unseen data. RF, NB, and GBC algorithms still had an acceptable level of AUC after validation. Additionally, the highest AUC was observed in the random forest, as shown in Figure S9.


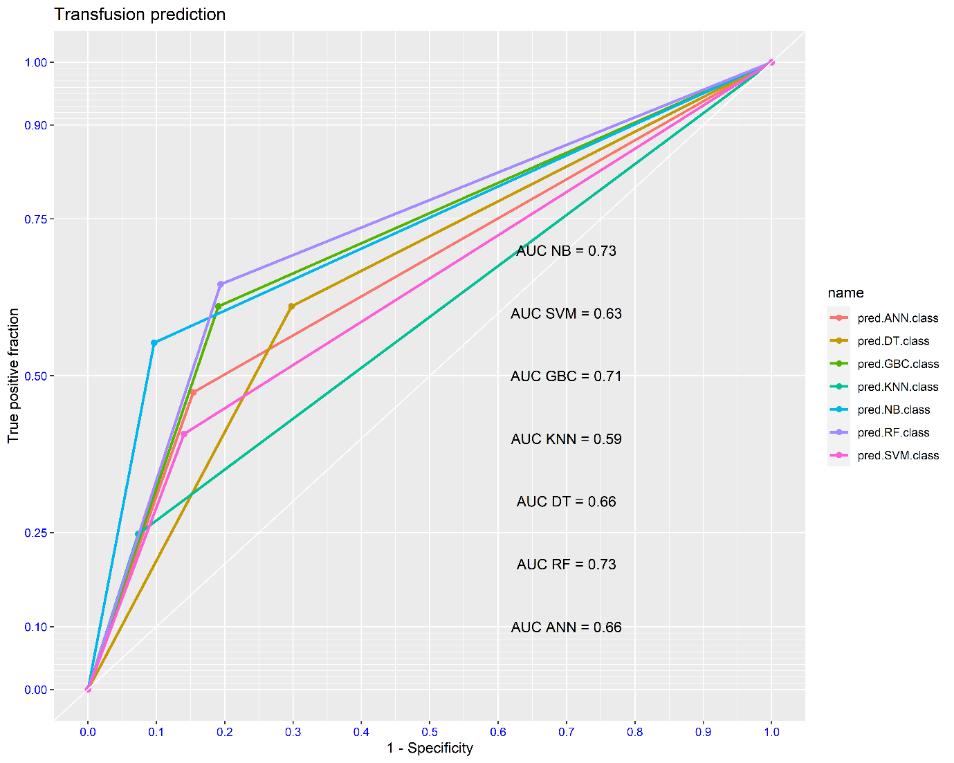


**Figure S9. The receiver operating characteristic curves with area under the curve of classification using 2^nd^ cohort. Abbreviations: ANN= artificial neural network, DT= decision tree, GBC= Gradient boosting classifier, KNN= k-nearest neighbors, NB= naïve Bayes, SVM= Support vector machine**

Moreover, the performance of PRC transfusion prediction by ML regression was present by scatter plots, as shown in Figure S10.


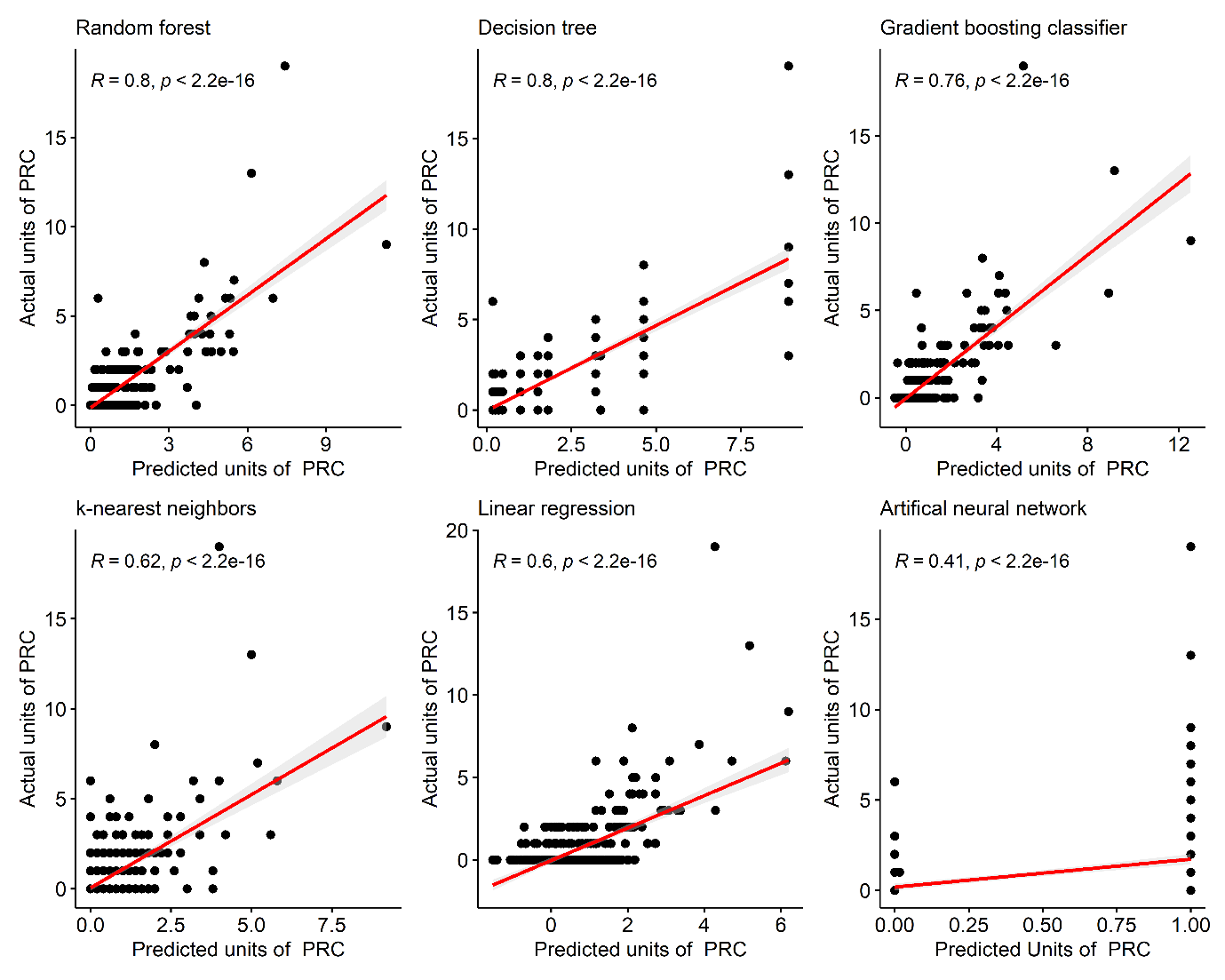


**Figure S10**. **Scatter plots of various regression algorithms for prediction of units of PRC using 2^nd^ cohort. Abbreviation: PRC= packed red cell, R= Pearson correlation.**

By ML regression, Table S9 shows results of intraoperative FFP transfusion validation. Liner regression had the lowest errors for validation.

**Table S9. Predictability of ML regression for intraoperative FFP transfusion by the 2^nd^ cohort (N=414)**

| **Algorithm** | **Pearson’s correlation** | **p-value of Pearson’s correlation** | **Spearman’s rank correlation** | **R^2^** | **RMSE** | **MAE** |
| --- | --- | --- | --- | --- | --- | --- |
| Linear regression | 0.87 | p<0.001 | 0.56 | 0.75 | 1.29 | 0.68 |
| Gradient boosting classifier | 0.87 | p<0.001 | 0.55 | 0.75 | 1.33 | 0.67 |
| Artificial neural network | 0.56 | p<0.001 | 0.63 | 0.31 | 2.41 | 0.94 |
| Decision tree | 0.87 | p<0.001 | 0.55 | 0.75 | 1.35 | 0.63 |
| Random forest | 0.87 | p<0.001 | 0.56 | 0.75 | 1.30 | 0.56 |
| k-nearest neighbors | 0.86 | p<0.001 | 0.54 | 0.73 | 1.42 | 0.66 |
| Abbreviations: FFP= fresh frozen plasma, R^2^= R-squared, RF= random forest classifier, RMSE= Root Mean Squared Error, MAE= Mean Absolute Error, ML=machine learning | | | | | | |

Additionally, scatter plots of various regression algorithms are presented using the 2^nd^ cohort, as shown in Figure S11.


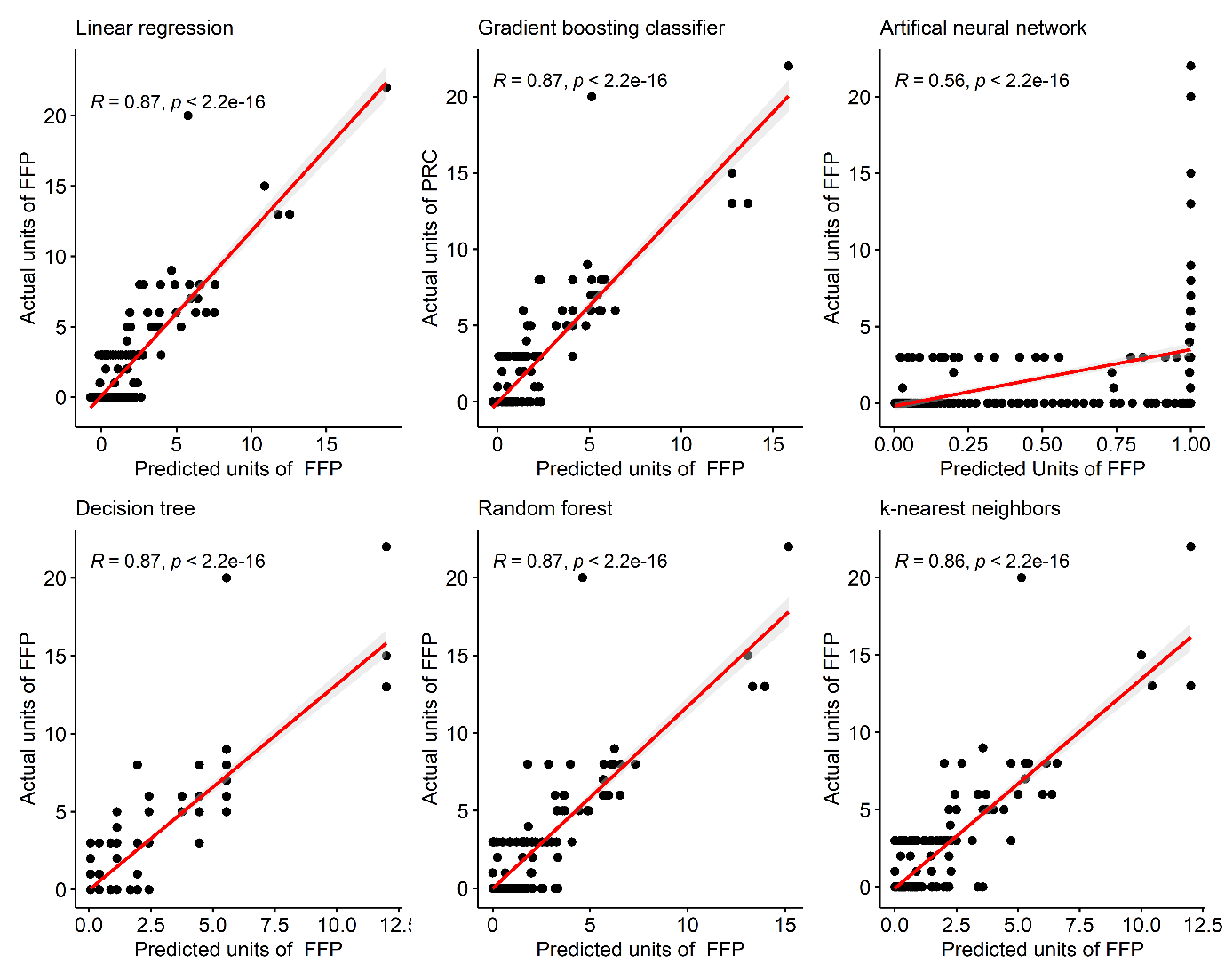


**Figure S11**. **Scatter plots of various regression algorithms for prediction of units of FFP using 2^nd^ cohort. Abbreviation: PRC= packed red cell, R= Pearson correlation.**
