# Supplementary material for: Economic impact of a machine learning-based strategy for preparation of blood products in brain tumor surgery
Source: PLoS One. 2022 Jul 1;17(7):e0270916. doi: 10.1371/journal.pone.0270916 (PMC9249218; doi:10.1371/journal.pone.0270916)
Supplement: Attachment — Submitted filename: Response to reviewer.docx [file pone.0270916.s003.docx]

**Response to reviewer**

| **Reviewer** | **Comment** |
| --- | --- |
| **Reviewer #1:** Economic Impact of a Machine Learning-Based Strategy for Preparation of Blood Products in Brain Tumor Surgery The main objective of this study was to identify the cost differences of the ML-based strategy compared with other strategies in preoperative blood products preparation.  For the reader it is hardly possible to compare the strategies. No lab parameters could be found as well as no evidence-based strategies. Therefore, the different strategies must be discussed in more detail. In some university hospitals it is allowed to take back packed red blood cells (pRBC) from the ward to the blood bank (established quality management system). This is not allowed in the US, but in Europe. Maybe you can talk with the responsible persons in your hospital. This must not be considered here.  ML is the strategy of choice and should be introduced wherever it is possible. Does it work also in catastrophes (Blackout)? Perhaps you can prepare another paper to answer this question. | Thank you for the valuable comments.  The ML-based model was built from several parameters that included preoperative laboratories, as shown in Supplement (Table S2, S5).  Several hospitals were allowed to return PRC from the ward to the blood bank if PRC did not be used for transfusion. However, the wasted time of cross-matched preparation and unnecessary workload should be managed. In the present study, ML-based strategy is one of the effective approaches to accomplishing over-requesting of preoperative blood preparation. This strategy had the lowest C/T ratio, and the highest Ti and Tp compared with other strategies that were discussed in line 221-223. |
| Introduction References 2, 3 of blood transfusion in neurosurgery in Thailand with limited meaningfulness. See for example: Curr Opin Anaesthesiol. 2014 Oct; 27(5): 470–473. doi: 10.1097/ACO.0000000000000109 McGirr, A, Pavenski, K, Sharma, B, Cusimano, M. Blood conservation in neurosurgery: erythropoietin and autologous donation. Can J Neurol Sci. 2014;41(5):583-589.  Meybohm, P., Fischer, D.P., Geisen, C. et al. Safety and effectiveness of a Patient Blood Management (PBM) program in surgical patients - the study design for a multi-centre prospective epidemiologic non-inferiority trial. BMC Health Serv Res 14, 576 (2014). <https://doi.org/10.1186/s12913-014-0576-3>  Goebel, BJA Patient blood management in intracranial neurosurgery—do we have sufficient data to define a transfusion threshold? Comment on Br J Anaesth 2018; 120: 988-98  No reference is given although it is used as supplementary data  Please, make an update of the literature | I revised the introduction and added the  citations as the reviewer’s suggestion. |
| Guidelines Reference 18, 19 are no guidelines, but clinical trials. Clinical Neurology and Neurosurgery, Volume 155, April 2017, Pages 83-89: Evidence-based outcomes for transfusion thresholds and indications are limited. Please, discuss the advantages and limits of your references in respect of missing guidelines. | I edited the errors as the reviewer’s suggestion. |
| Costs $3,0061.56, $57,313.92, and $136,292.94 Please, correct the error and use US-$. How did you calculate the costs? Since the prices of packed red blood cells are very different in different countries, I recommend giving percentages with reference to one of the basic methods. | How did you calculate the costs?  - It is the standard cost of our hospital.  That is the limitation of the study in generalizability because we used local parameters in the economic model.  However, this approach is the one of methods in the study of the economic evaluation that aim to reflect the economic problems of each country or region. For example, the cost-effectiveness of temozolomide (chemotherapy for glioblastoma) has been studied in several countries using their parameters and costs. [   - Messali A, Hay JW, Villacorta R. The cost-effectiveness of temozolomide in the adjuvant treatment of newly diagnosed glioblastoma in the United States. Neuro Oncol. 2013;15(11):1532-1542. doi:10.1093/neuonc/not096 - Waschke A, Arefian H, Walter J, Hartmann M, Maschmann J, Kalff R. Cost-effectiveness of the long-term use of temozolomide for treating newly diagnosed glioblastoma in Germany. J Neurooncol. 2018;138(2):359-367. doi:10.1007/s11060-018-2804-x - Wu B, Miao Y, Bai Y, et al. Subgroup economic analysis for glioblastoma in a health resource-limited setting. PLoS One. 2012;7(4):e34588. doi:10.1371/journal.pone.0034588]   Therefore, I further discussed this limitation in the revised manuscript in line 239-247.  Recommend giving percentages with reference to one of the basic methods.   - I added the percentages in Figure 4 and in line 196-197 as the reviewer’s suggestion. |
| Conclusions are too general The pandemic has caused a reduction in blood donation activity. Maybe this is true in Thailand, but not in other countries due to different rules to live with the pandemics. This general conclusion is not valid. It is an assumption, but not shown with data. It seems that partly the opposite is the case. ML-based strategy offers a way to calculate preoperative crossmatch and effectively reduce the cost of unnecessary blood products preparation via a web application. Cross match does not mean that the pRBC are on the ward and either transfused or lost if not needed. As long as the pRBC are in the refrigerater on ward or stil in the blood depot of the blood bank the pRBC can be used also for other patients. Please, formulate more precisely. | I edited the errors as the reviewer’s suggestion. |
| **Reviewer #2:** In these retrospective study the authors aimed to analyzed patient who received surgery from January 2014 and December 2021. Through the analysis of multiple preoperative parameters that may affect the RBC transfusion volume, they used ML algorithms to build up the artificial intelligence (AI) model to predict the accurate RBC demand quantity and compared each result with those predicted by clinicians and to evaluate the economic impact. Their results were shown that the ML methods is more accurate than clinician experience in predicting preoperative RBC transfusion, which reduce the unnecessary cost of blood preparation. This seems a promising study, can be accepted. | Thank you for the valuable comments. |
